# Enhancing bronchopulmonary dysplasia prediction in preterm infants using artificial intelligence and multimodal data integration

**DOI:** 10.3389/fped.2025.1629795

**Published:** 2025-12-04

**Authors:** Xinkai Zhang, Anping Wang, Rongwei Xu, Dongyun Liu

**Affiliations:** 1 Qingdao Medical College, Qingdao University, Qingdao, China; 2 Department of Pediatrics Medical Center, The Affiliated Hospital of Qingdao University, Qingdao, China; 3 MS in Computer Information Systems, Boston University Metropolitan College, Boston, MA, United States

**Keywords:** artificial intelligence, bronchopulmonary dysplasia, machine learning, multimodal data, predictive models

## Abstract

Bronchopulmonary dysplasia (BPD) remains a prevalent respiratory condition among preterm infants, with its development influenced by a combination of perinatal and postnatal factors. The development of artificial intelligence (AI) and machine learning (ML) technologies has provided new ideas for building BPD prediction models based on multimodal data (such as clinical information, physiological signals, imaging data, biomarkers, and omics data). This article systematically reviewed the research progress of AI in BPD prediction, analyzed representative models and key tools (such as RTI BPD Outcome Estimator), and assessed their performance and limitations in actual clinical settings. It also sorted out the challenges faced by AI models in clinical translation, including data standardization, model interpretability, system integration capabilities, model update mechanisms, and ethical and legal issues. To address the clinical need of “moving from prediction to intervention”, this article discussed the PALM translation framework (Predict–Act–Learn–Monitor) organized around key clinical nodes. In the future, it is necessary to strengthen multi-center data sharing, develop privacy protection technologies such as federated learning, and build a design, validation, integration, regulation, and feedback closed-loop management system to help AI models move from risk prediction to precise intervention, ultimately improving the clinical outcomes of children with BPD.

## 1 Introduction

Bronchopulmonary dysplasia (BPD) represents a significant respiratory condition frequently observed in preterm infants, with its incidence inversely associated with gestational age. Studies have shown that the incidence of BPD in extremely premature infants with a gestational age of 22–28 weeks is as high as 60.7% (1). The introduction and widespread implementation of antenatal corticosteroids (ACS) and pulmonary surfactant (PS) therapies have contributed to improved survival rates among extremely preterm infants—those with birth weights under 1,000 g and gestational ages below 28 weeks. However, this shift has also been accompanied by a rise in cases of “new BPD,” a phenotype marked by impaired alveolar development and diminished pulmonary microvascularization, with relatively milder alveolar and airway injury or fibrosis (2).

Infants with BPD frequently exhibit adverse long-term outcomes, including growth and developmental delays, as well as persistent impairments in cardiopulmonary and neurodevelopmental functioning (3, 4). In recent years, artificial intelligence (AI) has emerged as a transformative tool in healthcare, particularly in the analysis and management of complex, high-volume clinical data. This review provides a comprehensive overview of the application of AI and related technologies in the context of BPD prediction and management among preterm infants. Emphasis is placed on improving strategies for early identification and intervention, enhancing clinical decision-making, and supporting favorable long-term outcomes in this vulnerable population. Some previous reviews have addressed the ethical challenges and interpretability issues of AI models in the neonatal field (5, 6), but there is still a lack of a systematic assessment and framework analysis centered on BPD and integrated with specific clinical practice procedures. Therefore, this review, on the basis of summarizing the existing BPD prediction models, focuses on the key obstacles in their clinical translation process. At the same time, this article discussed the PALM translation framework (Predict–Act–Learn–Monitor), which is organized around key clinical nodes, with the aim of providing theoretical basis and practical reference for promoting the clinical implementability of BPD prediction models.

## 2 Overview of the research status of artificial intelligence in BPD prediction

### 2.1 Advantages of integration of multi-source data and AI models

In Neonatal Intensive Care Units (NICUs), preterm infants constitute a population particularly well-suited for AI-based monitoring of BPD. Currently, predictive models frequently incorporate physiological parameters such as heart rate, respiratory rate, oxygen saturation, and ventilator settings (7, 8). While NICU equipment facilitates continuous acquisition of large-scale physiological data, conventional monitoring techniques often lack the capacity to deliver accurate and real-time analyses due to the high variability of these indicators in preterm infants.

AI offers significant advantages in the prediction and management of BPD by enabling the integration and interpretation of complex, multidimensional clinical datasets. For instance, AI models embedded within electronic health record systems have been utilized to identify early risk signals for BPD from vital sign trends, ventilator metrics, and additional physiological variables. These models support clinical decision-making by contributing to early risk stratification, prognostic assessment, and the development of individualized treatment strategies (9, 10). Moreover, AI systems are capable of integrating diverse data sources, including imaging, genomic data, and biochemical profiles, to examine inter-organ interactions, thereby enhancing both the accuracy and comprehensiveness of predictive models (11, 12). The development of clinically applicable BPD risk assessment tools requires minimization of false-positive rates and maximization of clinical interpretability. It remains essential that healthcare professionals synthesize AI-generated outputs with comprehensive clinical evaluations to ensure that decision-making remains clinically grounded and contextually appropriate.

### 2.2 Pathway for AI model development and validation

In the context of BPD prediction, supervised learning is the predominant methodology. That is, based on the data with known labels, the model is trained to achieve the mapping between features and outcomes. In terms of application division of labor: structured clinical variables and laboratory data often adopt machine learning approaches (12–15). Medical imaging and high-dimensional continuous monitoring signals are more suitable for deep learning (13). During the model development phase, hyperparameter optimization is typically used to improve generalization performance. For model performance evaluation, in addition to AUROC (Area Under the Receiver Operating Characteristic Curve), it is recommended to report AUPRC (Area Under the Precision-Recall Curve) simultaneously to better reflect the ability to identify positive cases. Meanwhile, the Brier score is used to assess the consistency between probabilistic predictions and actual outcomes (16). The model validation phase mainly includes: internal validation (e.g., cross-validation, bootstrapping), which is used to evaluate consistency and robustness; external validation (using data from different institutions or time periods), which is used to test transferability across regions and systems (17). At present, most BPD-related AI models are still in the development or internal validation stage, lacking cross-center external validation and real-world data testing, which limits their feasibility for clinical application. Ultimately, evidence still relies on randomized controlled trials (RCTs) to assess their real-world benefits and safety for clinical outcomes, thereby supporting routine application.

### 2.3 Analysis of the progress of AI prediction models, tools and related literature

Currently, RTI BPD Outcome Estimator (https://neonatal.rti.org) is a widely used clinical tool for BPD prediction, featuring clear parameter settings, transparent logic, and a user-friendly interface, making it convenient for bedside rapid application (18). However, its limitation lies in that the model is constructed based on a small number of static variables (such as gestational age, birth weight, and oxygen concentration), and it is difficult to incorporate continuous physiological signals, medical images, and multi-omics data in the NICU. It also lacks a dynamic update mechanism and adaptive capabilities, which limits its adaptability in complex scenarios.

In recent years, the application of AI in BPD research has become increasingly widespread. Lei et al. used a random forest model with 6 variables to predict BPD, achieving an AUC of 0.929 (19). Dai et al. combined clinical and genetic information, and the AUC values for predicting BPD and severe BPD were 0.915 and 0.907, respectively—outperforming the model that used only clinical data (20). A study based on chest x-rays demonstrated that the AI model can accurately predict BPD within 24 h of birth, outperforming expert diagnosis (21); another transcriptomics study also showed that a small number of genes can effectively predict BPD, expanding the data dimension (22). However, a high AUC alone is insufficient to support clinical translation. First, in the context of class imbalance, AUC tends to be overestimated. It is recommended to report AUPRC simultaneously to reflect the value of identifying minority classes (patients with diseases). Second, features must be accessible at the time of prediction to avoid data/label leakage. Recent reviews and empirical studies have shown that leakage significantly exaggerates effect estimates, especially in small sample sizes (23); third, external validation must be completed across three dimensions—geography, time, and equipment—and the sample size, distribution of gestational age/body weight, definition of BPD, and its sensitivity analysis should be transparently disclosed; fourth, in terms of evaluation dimensions, the focus should expand from discrimination to calibration and clinical net benefit: beyond AUC/AUPRC, regularly report the Brier score, expected calibration error (ECE), and calibration slope/intercept, use Decision Curve Analysis (DCA) to quantify net benefits at preset adoption thresholds (24), and simultaneously provide the PPV (Positive Predictive Value), NPV (Negative Predictive Value), and alert rate at these thresholds to align with the action thresholds of NICUs; the aforementioned practices collectively enhance the model's robustness and adoptability, serving as a prerequisite for its entry into real-world evaluation and clinical trials.

## 3 Multimodal data sources supporting AI modeling: current status and integration potential

**Figure 1:**
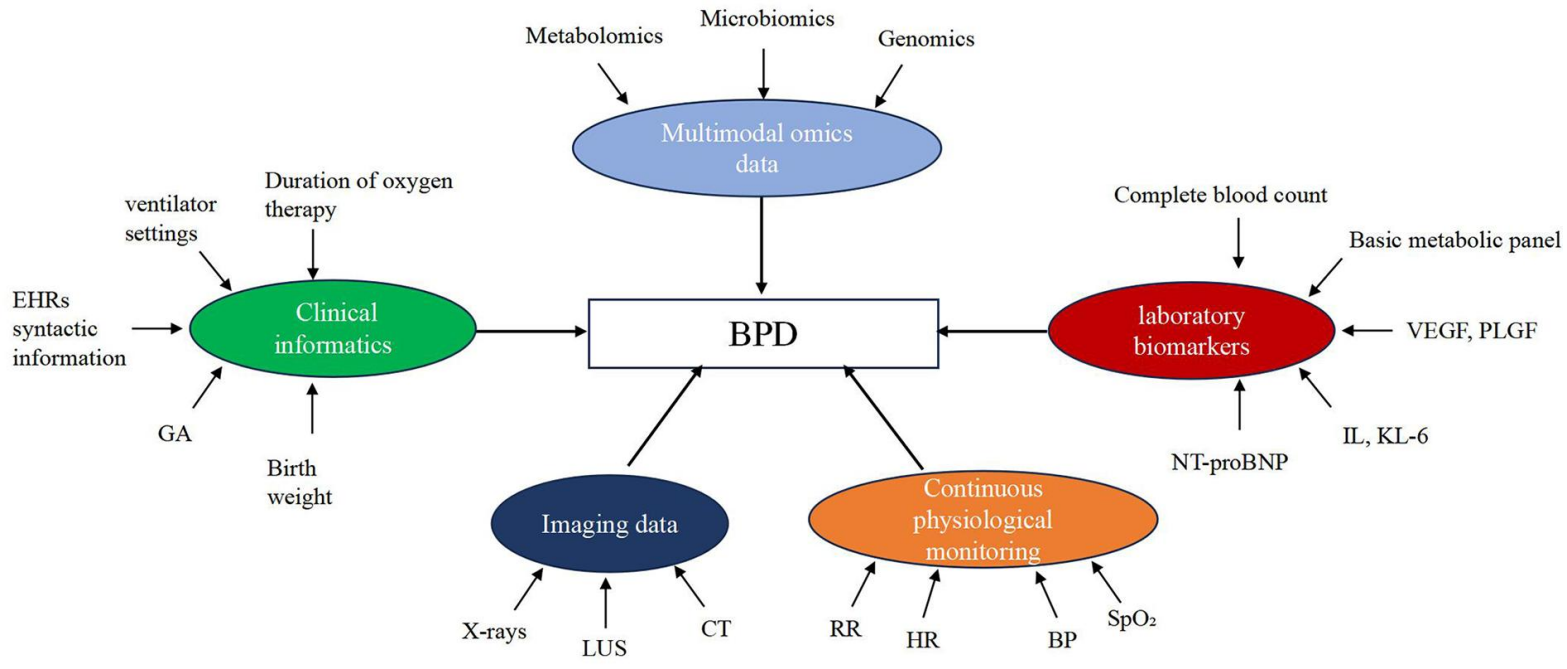
Multiple omics data sources used for modeling in BPD prediction by AI. BPD, bronchopulmonary dysplasia; EHRs, electronic health records; GA, gestational age; CT, computed tomography; RR, respiratory rate; HR, heart rate; BP, blood pressure; SpO₂, peripheral capillary oxygen saturation; VEGF, vascular endothelial growth factor; PLGF, placental growth factor; IL, interleukin; KL-6, Krebs von den Lungen-6; NT-proBNP, N-terminal pro-B-type natriuretic peptide; LUS, lung ultrasound.

The integration of diverse data modalities is anticipated to significantly enhance both the understanding and predictive accuracy of AI models for BPD. Figure 1 illustrates the connections between various data sources and BPD: clinical informatics (gestational age, birth weight, duration of oxygen therapy/ventilation, and structured EHR variables) provides patients' baseline information and disease progression context; continuous physiological monitoring (HR, RR, SpO_2_, and blood pressure) reflects dynamic changes in respiratory/circulatory status; imaging (CXR, LUS, and CT/MRI) offers objective signs of lung aeration and interstitial changes; laboratory biomarkers (inflammatory factors, KL-6, NT-proBNP, VEGF/PLGF, etc.) indicate inflammation, epithelial/interstitial damage, and pulmonary vascular load; and multi-omics (metabolomics, microbiomics, genomics/transcriptomics) reveal pathways and molecular-level mechanisms. On this basis, cross-modal synergy has clear practical value: the combination of imaging and physiological monitoring can be used for early warning and ventilation strategy optimization; the integration of physiological monitoring and laboratory biomarkers helps identify “re-elevation of inflammation-pulmonary vascular load,” guiding fluid management or pharmacological interventions (25); existing studies have shown that models integrating clinical information with multi-omics data yield better performance than single-modal models, suggesting that this direction has feasibility and scalable potential (20).

### 3.1 Clinical informatics data

BPD is recognized as a multifactorial disorder, and reliance on individual predictors is insufficient for accurate risk estimation, multivariate integration offers greater advantages (26). Observable clinical information corresponding to alveolar simplification is mainly reflected in the distribution and fluctuation of FiO₂ and SpO₂, the frequency and duration of intermittent hypoxemia (IH), and the oxygen saturation index (OSI). Phenotypes associated with increased pulmonary vascular load often manifest as persistently high oxygen demand under similar ventilation settings and difficulty in weaning from ventilation. In NICUs, high-resolution data (e.g., ventilator parameters, continuous SpO₂, blood gas results, OSI) and low-resolution data (e.g., demographics, medical history, medication records) together form longitudinal evidence, enabling early identification of ventilation-oxygenation mismatch and trends in respiratory work (27). Based on this, it is recommended to extract robust temporal features (median, variability, trend slope, etc.) from EHRs across three clinical time windows: ≤24 h after birth, 3–7 days after birth, and approximately 28 days after birth, with the features limited to those accessible at the time of prediction. The model output should provide calibrated individualized risk and report clinical net benefit under preset thresholds, facilitating direct alignment with ventilator parameter fine-tuning, oxygen therapy target setting, and weaning assessment.

It is important to emphasize that despite the richness of available clinical data, multiple challenges persist, including the labor-intensive nature of manual data entry, missing values, changes in disease definitions over time, difficulties in capturing complete longitudinal records, potential biases in data collection, and concerns related to data governance and privacy. These limitations underscore the necessity of advancing data acquisition and storage methodologies to enhance the robustness of BPD-focused research and model development.

### 3.2 The role of imaging and imaging AI in BPD

It complements the sequential variables such as EHR and enables repeatable quantification. In the NICU, chest X-ray (CXR) and bedside lung ultrasound (LUS) are the most accessible: CXR is convenient for longitudinal follow-up of lung gas volume and interstitial changes, while LUS can be frequently performed bedside and sensitively reflect lung gas volume and edema status (28). CT and MRI are more suitable for evaluating the pulmonary microstructure and ventilation/perfusion conditions in critical decision-making or research scenarios, but they are often limited by factors such as radiation exposure, time requirements, and the need for sedation (29).

The imaging AI technology is mainly divided into radiomics based on manual features and end-to-end deep learning. Usually, it needs to be combined with lung field segmentation, quality control and clinical variables for multimodal fusion to enhance the robustness and interpretability of the model (30). Research shows that the deep learning model based on neonatal CXR can output risk signals comparable to or even better than those of expert interpretations at an extremely early stage of birth. However, cross-center external validation is still insufficient, and it is urgently necessary to evaluate its generalizability under a unified annotation and standardized collection framework (21). Based on this, this article further proposes: The imaging AI evidence should be integrated into specific clinical time windows and intervention nodes, rather than just being a comparison of methodological performance. Thus, the experimental “imaging-AI” is transformed into a clinical pathway of “imaging-AI-guided intervention pathway”.

### 3.3 Structured biomarker data and the prospects of AI integration

Blood biomarkers are easy to standardize and structurally input, making them important potential input features for AI models. Existing evidence shows the following: Angiogenic factors (VEGF, PLGF) are crucial for pulmonary vascular and lung development (31). Serum VEGF/PLGF is expected to be used for early risk identification and intervention stratification (32). Inflammatory factors (IL-4/6/8, TNF-α, IL-33) reflect the persistence of inflammation and are associated with a high risk of BPD (33, 34). Systemic immune-inflammatory indices (e.g., NLR, SIRI) are significantly elevated at birth and at 36 weeks of gestational age in infants with moderate-to-severe BPD, indicating an increased systemic inflammatory load (35, 36). Among lung injury biomarkers, KL-6 indicates alveolar epithelial and pulmonary interstitial damage (37) and its level at 14 days after birth shows good discriminative power for BPD (AUC = 0.88) (38). NT-proBNP reflects volume load/pulmonary vascular stress (39); in very low birth weight infants, NT-proBNP > 2.264 ng/L at 14 days after birth can early indicate the risk of BPD (sensitivity: 100%, specificity: 86%, AUC = 0.93) (40).

For clinical application, biomarker modeling should align with disease course time windows and pathological pathways: Within ≤24 h after birth, prioritize indicators reflecting alveolar/interstitial damage and early inflammation (e.g., KL-6, IL-6/IL-8) for extremely early risk stratification; from 3 to 7 days after birth, combine NT-proBNP with inflammatory profiles to identify the possible “re-elevation of inflammation-pulmonary vascular load,” so as to indicate the timing of respiratory support and fluid/pharmacological intervention; at approximately 28 days after birth, integrate persistent oxygen dependence, nutritional/growth indicators, and VEGF/PLGF to assess phenotypes of persistent damage and microvascular development disorders and plan follow-up. Integration with other modalities should follow the principle of “starting with the easy and moving to the difficult”: When sample size is limited or modalities are frequently missing, adopt late fusion (train each modality independently, then perform confidence weighting or stacking integration after outputting calibrated probabilities)—this facilitates external validation and allows the use of Decision Curve Analysis (DCA) to demonstrate clinical adoptability; after multi-center data and annotations are improved, transition to mid-level fusion (jointly model “pathway-level” biomarkers with imaging and temporal physiological data within the same time window) to obtain cross-modal information gain and mechanistic interpretability (41).

Although the above studies mostly employed traditional statistical models, their data structure is highly suitable for being incorporated into AI systems for multi-feature modeling. Although the blood biomarkers have shown certain potential initially, due to the small sample size of the research and the high clinical heterogeneity, they are currently still in the research stage and have not yet entered the routine clinical application process. Larger-scale multi-center studies are needed to verify their efficacy (36).

### 3.4 A large number of biological information data and future AI modeling directions

Multi-omics provides a key supplement for revealing the molecular and pathway-level mechanisms of BPD. At the metabolomics level: Premature infants with BPD show elevated levels of alanine, serine, taurine, and citrulline in bronchoalveolar lavage fluid on the first day after birth (42). Meanwhile, studies have shown that the levels of hydroxy fatty acids and oxidized fatty acids in the amniotic fluid of premature infants with BPD are higher than those in the non-BPD group (43, 44). At the microbiomics level: Research indicates that compared with the non-BPD group, the pulmonary microbial community of the BPD group shows a trend of increasing proportion of Firmicutes and decreasing proportion of Proteobacteria over time (45). Additionally, gut microbiota may affect BPD development through modulation of systemic immunity and inflammatory responses.

At the genomics/transcriptomics level: Variations in angiogenesis pathways (e.g., VEGF) and abnormalities in airway exosomal miRNAs (such as miR-876–3p, miR-20a-5p) are associated with BPD susceptibility (43, 46).

To advance omics evidence from “description” to “computational evidence usable for prediction and stratification,” two types of alignment need to be achieved. First is methodological alignment: perform batch effect correction and scale unification for multi-center/multi-platform data, and reduce high-dimensional omics features to pathway/module-level representations (e.g., aggregating genes/metabolites into activity scores for pathways such as “inflammation, oxidative stress, and angiogenesis”) to lower dimensionality and facilitate mechanism-based interpretation. Second is temporal alignment: align omics data with concurrent imaging data and continuous physiological signals according to clinical prediction windows (0–24 h, 3–7 d, ≈28 d) for joint modeling, ensuring features are accessible at the time of prediction and avoiding data/label leakage.

For integration strategies, progression should follow the principle of “late fusion first, then mid-level fusion.” When sample size and modality accessibility are limited, late fusion should be adopted first. As multi-center samples and annotation standards are gradually refined, transition to mid-level fusion: jointly characterize pathway-level omics, imaging, and other data within the same time window, and use multi-modal Transformers (with cross-modal attention and mask training to enhance robustness to missing modalities) or heterogeneous graph neural networks (constructing graphs of “gene/metabolite/microbiota—pathway—phenotype” and performing message passing for importance attribution) to improve mechanistic interpretability (47, 48). Existing small-sample prototypes have shown that combined metabolomic-microbiomic multi-omics exhibit cross-modal consistent signals in inflammation/oxidative stress pathways (49). However, regardless of the approach, multi-omics integration can only be transformed into interpretable, verifiable, and implementable decision support in NICU settings if discrimination, calibration, net benefit, and robustness all meet the required standards.

## 4 Analysis of the actual obstacles in the clinical application of AI prediction models

### 4.1 Data-related limitations

The implementation of DL models within clinical settings necessitates seamless integration with healthcare information systems, including EHRs and imaging databases. However, this process often encounters challenges such as high technical barriers, long implementation periods, large capital investment, and complex compliance requirements. Especially in developing regions with lower levels of informatization, the data foundation is weak, and obtaining high-quality samples is limited. Moreover, the training data has insufficient representativeness for some populations, all of which affect the generalization performance of the model (50). Furthermore, medical records often suffer from missing information and incorrect input. The data structures from different sources such as EHR, wearable devices, and medical images vary greatly, making it difficult to integrate across institutions, which undermines the stability and accuracy of the model (51–54). Currently, the related research on BPD lacks open and shared datasets and code libraries, which limits the verification of results and the re-development of models, and hinders its generalizability across different institutions.

### 4.2 Model-related limitations

1. **Lack of interpretability (“Black Box” issue):** Deep learning-based AI models often function as “black boxes” due to their inherent complexity and opaque decision-making mechanisms. The lack of transparency may undermine clinician trust, particularly in critical clinical decisions such as treatment discontinuation or experimental interventions, where comprehensible rationale is essential for adoption (55).
2. **Insufficient generalization ability and external validation:** AI models are susceptible to overfitting, wherein they capture noise or irrelevant patterns specific to the training dataset. While such models may exhibit high accuracy in controlled experiments, their application is ineffective in different populations or medical systems. The prevalence, manifestations and data sources of BPD are greatly influenced by factors such as race, gestational age, and socioeconomic status, which limits the universality of the model (56). At present, most models lack systematic external validation studies, especially cross-institutional and multi-center data validation is rare, and their applicability in real-world environments cannot be confirmed.
3. **The model update mechanism is not perfect:** Clinical data and disease spectra have dynamic characteristics. If the model lacks a real-time monitoring and update mechanism, it is prone to “model drift”, that is, changes in the distribution of input data lead to performance degradation (57). Ideally, an AI model should have the capabilities of continuous performance monitoring, drift detection, and automated re-training, forming a closed-loop feedback system. Currently, most BPD prediction models lack drift monitoring mechanisms, as well as no standardized procedures for model re-training or version iteration, making it difficult to ensure their long-term stability and clinical applicability (58).

### 4.3 Clinical integration and ethical supervision aspects

1. **The integration and transformation path of clinical applications are unclear:** Most BPD AI models are limited to research or tool levels and have not been deeply coupled with treatment paths and intervention processes, making it difficult to guide actual clinical behaviors. Currently, there is a lack of standardized prediction, stratification, intervention processes, and operational guidelines, which limits their practical application in the clinical setting.
2. **Ethical compliance and regulatory mechanisms are lagging behind:** When AI is applied to the prediction of neonatal diseases, it involves sensitive populations, and its data usage and model deployment must strictly comply with ethical norms. Currently, there is a lack of specialized ethical guidelines and data regulatory mechanisms for pediatric AI models. Issues such as informed consent, data anonymization, algorithm bias correction, and liability attribution for model failures all urgently require clear legal and institutional definitions (59).

## 5 From prediction to intervention: building an AI-driven clinical intervention path for BPD

### 5.1 Establishing a “time window–PALM” translation framework for NICUs

Currently, the research on AI in BPD management mainly focuses on risk prediction, but there is less discussion on how to convert the prediction results into specific intervention measures (60). Based on existing systematic evidence, this review proposes a Time Window–PALM (Predict–Act–Learn–Monitor) framework, which takes ≤24 h, 3–7 days, and ≈28 days after birth as key nodes in the disease course, requires the model to output calibrated individualized risk at each node and use Decision Curve Analysis (DCA) to provide net benefits under acceptable thresholds, and maps risk thresholds to specific intervention measures (e.g., ventilator fine-tuning, oxygen therapy targets, medication/fluid management, imaging re-evaluation); within fixed time windows, the following should be routinely reported: calibration and net benefit, alert rate, and robustness across subgroups (e.g., by gestational age or center), which are used to trigger or delay interventions, and subsequently, real-world outcomes are fed back to recalibrate the model and conduct incremental update training while continuous monitoring of model performance and drift is required to ensure traceability and auditability.

### 5.2 Individualized treatment intervention strategies at different stages

The clinical value of AI lies in achieving refined, phased, and individualized treatment guidance (6). Within the first 24 h after birth, the model provides calibrated risk, net benefits at selected thresholds, and simultaneously presents PPV, NPV, and alert rate; if the risk exceeds the center's threshold, parameter optimization for lung-protective ventilation is first implemented (limiting tidal volume within the allowable oxygenation/carbon dioxide targets, setting appropriate PEEP, and reducing high-pressure impact), and lung recruitment strategies and oxygen therapy targets are reviewed based on CXR/LUS, followed by re-evaluation of risk trajectory and adverse events at 24–48 h to verify the immediate effect.

From 3 to 7 days after birth, calibrated individualized risk assessment is updated under the same methodological framework and interpreted in conjunction with inflammatory profiles/cardiopulmonary load signals (e.g., CRP/IL-6/IL-8, NT-proBNP, LUS scores); when both risk and net benefits meet the standards, stratified medication and fluid management can be initiated: for infants with severe inflammatory phenotypes and weaning difficulties, glucocorticoid regimens are selected based on evidence, while infants with suspected increased fluid/pulmonary vascular load receive fluid restriction/diuresis and supportive therapy, with re-testing at 48–72 h to evaluate efficacy and safety.

At approximately 28 days after birth, if infants still have persistent oxygen dependence, longitudinal oxygen demand curves, weight gain/nutrient intake, KL-6/NT-proBNP, and imaging features are comprehensively interpreted to distinguish phenotypes dominated by interstitial damage/alveolar simplification or accompanied by significant pulmonary vascular load: the former prioritizes nutritional and antioxidant support, rehabilitation, and weaning assessment, while the latter undergoes multi-disciplinary cardiopulmonary evaluation and necessary advanced imaging/functional tests, with the establishment of a long-term follow-up plan.

To facilitate understanding of practical implementation, two contextual examples are provided below: A very preterm infant was identified as high-risk by the model 6 h after admission, with positive threshold net benefits shown by DCA; the team accordingly adjusted down ventilator parameters and calibrated oxygen therapy targets, and re-evaluation at 48 h showed decreased OI/OSI and controllable alert rate, so the intervention was retained and documented. On day 5, NT-proBNP increased and LUS score worsened, with risk exceeding the threshold again; short-course low-dose glucocorticoids and fluid restriction were administered in accordance with prescription guidelines, and re-evaluation at 72 h determined whether to upgrade imaging or discontinue pharmacological intervention.

### 5.3 Operational key points and required resources for the PALM closed-loop

Predict: Use features accessible at the time of prediction, and routinely report calibration and threshold ranges indicated by DCA; it is recommended to centrally establish time-windowed risk control tables (including: risk, threshold, net benefit, preset actions, and re-evaluation time points) (61).

Act: Solidify “threshold-action” into executable medical orders/pathways (ventilator parameter targets, oxygen therapy ranges, recruitment strategies, inclusion/exclusion criteria for glucocorticoid and fluid management); run in parallel (shadow mode) in the initial launch phase to prevent premature automation (62).

Learn: Conduct incremental/periodic retraining and threshold recalibration based on real-world outcomes; supplement with feature ablation and key subgroup reviews to maintain stability (62).

Monitor: Continuously monitor performance, fairness, and drift; retain audit logs and version control; align with incident reporting-rollback processes, as well as information notification and appeal channels for family members and medical staff to ensure traceability and accountability (63).

## 6 Future research prospects

Although the application of artificial intelligence (AI) in the risk prediction and individualized management of bronchopulmonary dysplasia (BPD) has achieved initial results (6, 12). However, its clinical application still faces multiple challenges such as data, models, ethics, and clinical pathways. Future research can focus on the following key directions:

First, a multi-center, high-quality clinical database should be established. Currently, most models are based on single-center data, which is limited by sample size and population heterogeneity, resulting in weak generalization ability. Efforts should be made to establish structured clinical databases covering multiple regions, multiple ethnic groups, and multiple NICUs. The data format and label system should be unified to provide a data foundation for model development, external validation, and multimodal integration, and to promote algorithm openness and research reproducibility.

Second, enhance model transparency and interpretability. Black-box models limit doctors' trust and willingness to adopt. In the future, interpretable artificial intelligence (XAI) technologies should be introduced (64) to provide visualizations of the contributions of clinical variables and tracking of decision paths, enhancing the clinical reviewability and adoption intention of the model.

Third, promote the deep integration of the model with the clinical system. The AI model should seamlessly integrate with clinical information system (HIS/EHR) through standard interfaces, automatically extracting key variables (such as gestational age, ventilator parameters, oxygen saturation, blood gas indicators, etc.) to enable real-time prediction and risk alerts (65–68). At the same time, a dynamic update mechanism should be established to adapt to data drift and changes in the clinical environment, ensuring the long-term stable operation of the model. For example, a periodic re-training mechanism and an automatic warning system should be introduced to build an “adaptive model update platform”.

Fourth, develop individualized prediction and mechanism-driven models. The development process of BPD is time-dependent, and it is suitable to adopt time series models to integrate continuous physiological signals (69–71). This enables dynamic identification of the disease progression trend. To reduce the impact of model drift in the BPD intervention path, it is necessary to regularly collect the latest data of BPD children for re-training (72). A real-time data monitoring and performance warning mechanism can be established, and the deviation between the input data and the initial training set can be analyzed regularly. To improve the model's adaptability, XAI tools such as SHAP can be introduced to trace the reasons for drift, clarify the reasons for changes in feature contributions, and optimize the individualized intervention strategies of the clinical path in a targeted manner. In addition, integrating multi-omics data (such as transcriptome, proteome, microbiome, etc.) will help identify the biological subtypes of BPD (73), it helps to identify biological subtypes and promotes precise prediction and the formulation of treatment paths.

Fifth, improve ethical governance and regulatory mechanisms. The clinical application of AI models must comply with ethical and legal norms. Data security can be protected through technologies such as differential privacy and federated learning (74), to clarify the ownership and responsibility of data usage. At the same time, establish an AI ethics approval and supervision mechanism for newborns to ensure their compliant and safe application in clinical settings.

## 7 Discussion

The advancement of AI within the medical domain has highlighted its increasing potential in the prediction and management of BPD among preterm infants. The present review has provided a comprehensive evaluation of AI applications in the development of BPD prediction models, including aspects such as multimodal data integration, model architecture and refinement, performance assessments of existing models, and limitations related to clinical implementation. Collectively, AI offers innovative tools to facilitate the early detection and individualized management of BPD. However, the practical application of these tools remains constrained by several unresolved challenges, necessitating ongoing interdisciplinary collaboration and model refinement.

Currently, a substantial proportion of AI-based BPD prediction models are limited to internal validation, with minimal utilization of external datasets for independent evaluation. Given the considerable variability in neonatal care practices, data acquisition protocols, and infant characteristics across NICUs, model generalizability and applicability require verification through external validation. Consequently, the establishment of a broad, multi-center collaborative framework is imperative. Such a network would support the harmonization of data standards and facilitate data sharing, thereby enhancing the diversity and representativeness of training datasets and strengthening the reliability of predictive models in diverse clinical contexts.

Ethical and legal issues must be considered in the clinical promotion of AI models. AI models typically rely on personal data, such as health records, hematological biomarkers, imaging data, and multimodal data, including genomics. As the volume of these data continues to increase, particularly when integrating multiple data sources and developing new ones, privacy concerns become more prominent (75). However, overly stringent privacy protections during data collection, use, and sharing may impede the potential benefits that patients could derive from using this data to advance AI-driven solutions, such as new diagnostic or treatment options (75, 76). Emerging methods, such as generating synthetic data through AI-generated samples, offer a way to protect privacy while fostering innovation. This synthetic data can be shared securely and used to develop new models (77). Additionally, legal disputes persist regarding the ownership and usage rights of health data, with current regulations failing to clearly define the ownership of AI training data. Therefore, prior to the clinical application of AI models, it is essential to establish comprehensive safety assessments and accountability mechanisms.

The Good Machine Learning Practice (GMLP) proposed by the FDA/IMDRF outlines 10 full-lifecycle principles. The EU's AI Act classifies AI for medical use as a high-risk system, requiring the establishment of lifecycle-wide risk management, high-quality data governance, transparency and user information, human oversight, as well as post-market monitoring and traceable documentation—all of which align with the NICU's goal of protecting minors (78). For pediatric scenarios, the ACCEPT-AI framework and UNICEF's AI Policy Guidelines for Children can be integrated to implement child-centered rights protection.

Under this framework, this study proposes three categories of actionable solutions:

Informed consent adopts a “stratified + dynamic” model (signed by parents/guardians as proxies, with reviews at admission, discharge, and follow-up nodes). The consent document must define the purpose and secondary use of data, withdrawal procedures and timelines, and implement “minimum necessary collection” and de-identification.

Bias auditing runs through both data and models: assess representativeness by gestational age, weight, gender, and region/center; report calibration and subgroup performance simultaneously during internal and external validation, and use Decision Curve Analysis (DCA) to present net benefits at different thresholds; develop plans for regular fairness reviews and bias correction.

For accountability and operational governance, a three-line responsibility system consisting of “clinical lead + data protector + engineering lead” is implemented: complete model and Data Protection Impact Assessment (DPIA) before launch; first run in shadow mode (model operates in parallel with manual processes without directly issuing medical orders) and gradually integrate with “gating thresholds”; during operation, conduct drift monitoring, retain audit logs and version management, and establish incident reporting-rollback channels as well as explanation and appeal channels for family members and medical staff.

In recent years, various AI models related to BPD have emerged continuously, but the ones that have truly achieved clinical routine application are still very limited. Most AI medical models are still at the development and preliminary validation stage, and only a very few can pass strict external validation. Moreover, the models that can be deployed in clinical settings or undergo prospective clinical trials are even more limited (79–81). For instance, a systematic review included 65 studies, covering 158 developed models and 108 externally validated models. Although some models have good discrimination, they generally have high bias risks, and most lack external validation and calibration assessment, which limits their clinical application (81). Although the French EPIPAGE-2 cohort has undergone cross-border validation, its predictive ability has reached a relatively high level (c-statistic 0.80–0.81), yet due to regional calibration deviations, it is still difficult to be generalized to real clinical settings (82).

To promote the effective transformation of AI models in the clinical management of BPD, it is suggested that the clinical needs be incorporated into the model development process to construct an interpretable and integrable model architecture. In terms of data, a multi-center shared database should be established to promote the standardization of data collection, storage and exchange, and enhance the generalization and reproducibility of the model (83). After deployment, a model performance monitoring mechanism should be set up and a dynamic update strategy should be adopted to deal with “model drift”. In terms of technical implementation, privacy-preserving computing (such as federated learning) can be introduced to facilitate cross-institutional collaboration. In terms of regulation, a unified AI medical regulation system has not yet been established globally, and only some countries have explored preliminary paths. It is recommended to draw on the management ideas of the FDA regarding AI medical software (74, 84). The supervision should cover model approval review, performance assessment, ethical review, continuous monitoring and exit mechanism, to ensure the safety, effectiveness and fairness of the model at all stages of clinical application. It is suggested that the national health administrative department take the lead in supervision, and collaborate with interdisciplinary experts from ethics, law, information technology, etc., to establish a “Medical AI Safety Assessment Platform”, formulate unified supervision norms, and achieve cross-institutional and cross-regional collaborative supervision.

The application of AI in BPD prediction has demonstrated considerable potential; however, its clinical translation still faces several challenges, including poor model interpretability, insufficient data quality, and inadequate ethical regulations. Moving forward, AI technology can effectively assist physicians in identifying high-risk infants at an early stage and optimizing intervention strategies, thereby improving both the quality of survival and long-term prognosis for premature infants, provided there is collaborative effort across technical, ethical, and institutional domains.

## 8 Conclusion

BPD is a prevalent and serious respiratory disease among premature infants, artificial intelligence technology provides a brand-new perspective for its early identification and precise intervention. This review systematically summarizes the research progress of BPD prediction models from aspects such as AI modeling principles, representative tools, clinical transformation obstacles and future directions. It focuses on the critical comprehensive evaluation of AI methods and clinical tools, highlighting core challenges such as model transparency, data standardization, system integration and ethical supervision, at the same time, this article discussed the PALM translation framework (Predict–Act–Learn–Monitor), which is organized around key clinical nodes. Future research needs to focus more on the deployment adaptability of AI models in real clinical environments, and build a complete transformation chain covering model design, verification, integration, regulation and feedback. To this end, priority should be given to promoting the construction of multi-center standardized databases, developing AI tools with interpretability and dynamic update capabilities, exploring privacy protection mechanisms such as federated learning, and developing closed-loop intelligent systems that can be embedded in clinical processes. This will truly achieve the transformation from “risk prediction” to “precise intervention”, and ultimately improve the long-term health outcomes of premature infants with BPD.
